# *In Vivo* Screening for Secreted Proteins That Modulate Glucose Handling Identifies Interleukin-6 Family Members as Potent Hypoglycemic Agents

**DOI:** 10.1371/journal.pone.0044600

**Published:** 2012-09-04

**Authors:** Chen Amy Chen, Peter C. Carolan, Justin P. Annes

**Affiliations:** 1 Department of Stem Cell and Regenerative Biology, Harvard University, Cambridge, Massachusetts, United States of America; 2 Department of Medicine, Massachusetts General Hospital, Boston, Massachusetts, United States of America; 3 Department of Medicine, Stanford University Medical School, Stanford, California, United States of America; University of Bremen, Germany

## Abstract

Diabetes is a disease of abnormal glucose homeostasis characterized by chronic hyperglycemia and a broad array of consequent organ damage. Because normal glucose homeostasis is maintained by a complex interaction between behavior (feeding and physical activity) and metabolic activity that is modulated by inter-organ signaling through secreted factors, disease modeling *in vitro* is necessarily limited. In contrast, *in vivo* studies allow complex metabolic phenotypes to be studied but present a barrier to high throughput studies. Here we present the development of a novel *in vivo* screening platform that addresses this primary limitation of *in vivo* experimentation. Our platform leverages the large secretory capacity of the liver and the hepatocyte transfection technique of hydrodynamic tail vein injection to achieve supraphysiologic blood levels of secreted proteins. To date, the utility of hydrodynamic transfection has been limited by the deleterious impact of the variable transfection efficiency inherent to this technique. We overcome this constraint by co-transfection of a secreted luciferase cDNA whose product can be easily monitored in the blood of a living animal and used as a surrogate marker for transfection efficiency and gene expression levels. To demonstrate the utility of our strategy, we screened 248 secreted proteins for the ability to enhance glucose tolerance. Surprisingly, interleukin-6 and several of its family members but not other well-recognized insulin sensitizing agents were identified as potent hypoglycemic factors. We propose this experimental system as a powerful and flexible *in vivo* screening platform for identifying genes that modulate complex behavioral and metabolic phenotypes.

## Introduction

Type 2 diabetes (T2DM) is a metabolic disease pandemic that affects more than 347 million individuals worldwide and is characterized by abnormal glucose homeostasis and consequent multi-organ damage [1], [2]. Unfortunately, current behavioral and pharmacological treatments are not very effective at reducing hyperglycemia in the long-term. While lifestyle interventions targeting diet and exercise typically have high relapse rates, pharmacological treatments fail to prevent the decline in β-cell function associated with disease progression in the majority of patients [3]–[5]. Thus, there is an urgent need for effective T2DM therapeutics.

The pathophysiologic hallmark of T2DM is obesity-related loss of insulin potency (peripheral resistance), which leads to an increased insulin-demand that ultimately exceeds the insulin-producing capacity of islet β-cells [6], [7]. One therapeutic strategy for T2DM is to restore insulin sensitivity by augmenting or inhibiting endogenous signaling pathways that enhance or impair insulin activity, respectively. To date, several hormones/cytokines that impact glucose homeostasis have been identified. These factors are produced by several different tissues including pancreatic islets (amylin, glucagon, insulin), adipocytes (adiponectin, leptin), hepatocytes (FGF21), intestinal endocrine cells (GLP-1), myocytes (IL-6) and inflammatory cells (TNF-αIL-1β) [8]–[16]. Indeed each of these factors is considered to be of therapeutic relevance. Perhaps furthest along in development are GLP-1-derived agents, which improve glucose tolerance by enhancing insulin secretion, increasing satiety, decreasing gastric emptying, and lowering glucagon secretion when they are given pharmacologically [17].

Given the complex multi-organ nature of glucose homeostasis, it is likely that additional factors that influence whole-organism physiology and improve insulin potency remain to be discovered. This speculation is supported by the major limitations of existing screening approaches that might be used to discover endogenous hypoglycemic factors. Whereas *in vitro* screening fails to take into account the complex interactions between an animal's behavior and inter-organ signaling, *in vivo* screening presents a barrier to moderate throughput studies. Typically, the feasibility of *in vivo* screening is limited by the time-intensive and expensive production of viruses for transfection and the variable transfection efficiency of established techniques such as retroviral integration [18], [19]. To overcome these limitations, we have developed an *in vivo* screening platform that allows secreted factors to be quickly, easily, reliably, stably, and inexpensively expressed in living animals. Here we present our efforts to identify secreted factors that enhance glucose tolerance via the establishment of a novel *in vivo* screening platform. Surprisingly, our studies identify interleukin-6 (IL-6) and several of its family members as potent hypoglycemic agents.

## Results

### Hydrodynamic tail injection mediated gene expression in hepatocytes

To develop a moderate throughput *in vivo* assay for secreted proteins that modulate glucose homeostasis, we required an efficient system for the delivery and expression of multiple secreted proteins in mice. Prior work has demonstrated that variable but stable hepatocyte transfection is achieved using hydrodynamic tail vein injection combined with the *Sleeping Beauty* (SB) transposon system [20]–[22]. Hydrodynamic tail vein injection is a technique that forces hepatocytes to take up naked DNA by rapidly delivering a large volume of DNA-containing solution into the tail vein of a mouse and generating elevated hydrostatic pressure within the liver [23]. The SB transposon system may be used in conjunction to promote the stable genomic DNA integration of expression constructs that are flanked by the *Sleeping Beauty* transposable element. Integrated expression constructs allow for stable hepatocyte expression of any gene of interest, including secreted factors which have the capacity to act on distant target tissues.

**Figure 1:**
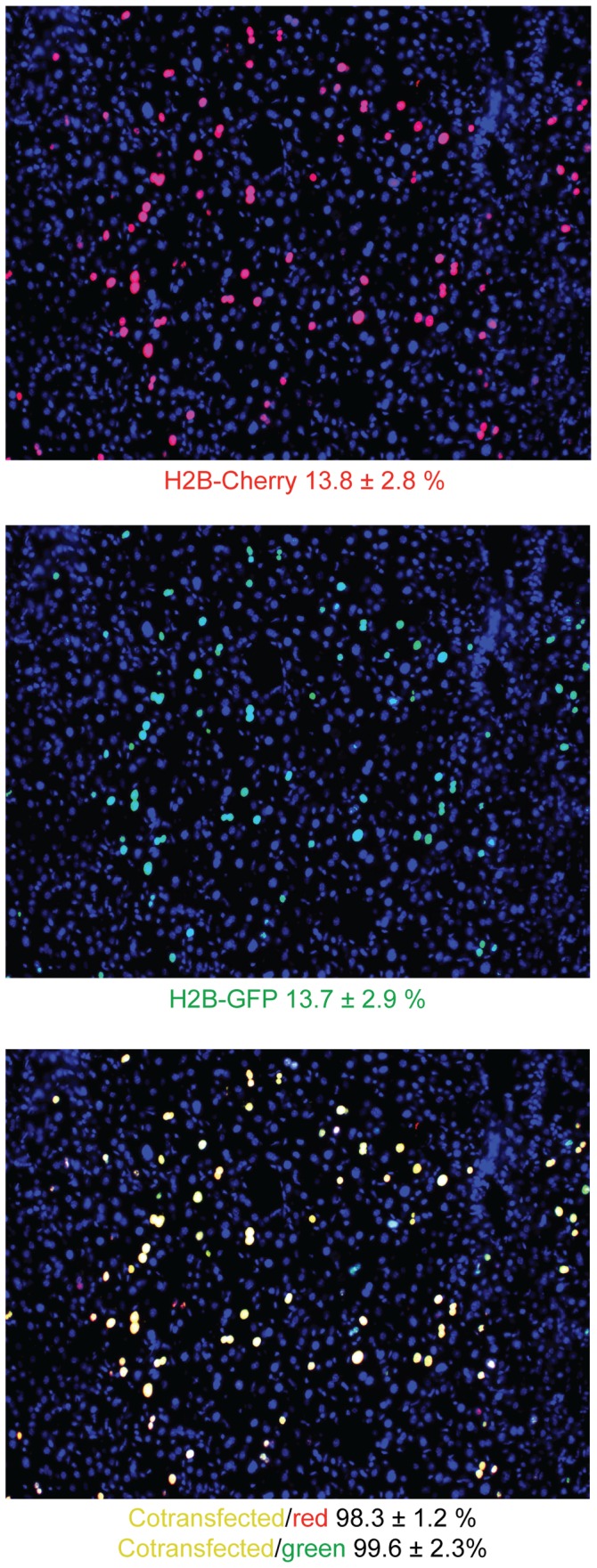
Hydrodynamic tail injection allows for hepatocyte co-transfection of multiple genes. Tail vein injections were performed on 6-week old ICR mice with 250 µg of an H2B-Cherry transposable expression plasmid (top panel) and 25 µg of an H2B-GFP transposable expression plasmid (middle panel). The percentage of individually transfected and co-transfected hepatic cells was determined by image analysis of fixed hepatic sections. Co-localization was identified by the co-localization of red and green fluorescence (bottom panel). Greater than 1000 cells from multiple sections were counted from a representative animal. Standard deviations are shown.

To assess the efficacy of hydrodynamic tail injection as a gene delivery tool for multiple cDNA constructs, we co-injected animals with a mixture of H2B-cherry and H2B-GFP expression plasmids at a molar ratio of 10∶1 and determined (1) the percentage of transfected hepatocytes with each construct and (2) the co-localization of the expressed fluorescent proteins. Image analysis of liver sections from a successful tail vein injection showed that 13.8%±2.8% of hepatocytes were H2B-cherry positive (Fig. 1, left panel) and 13.7%±2.9% of hepatocytes were H2B-GFP positive (Fig. 1, middle panel). Of all H2B-cherry positive cells, 98.3±1.2% were co-transfected with H2B-GFP, and of all H2B-GFP positive cells, 99.6%±2.3% were co-transfected with H2B-cherry (Fig. 1, right panel). We note that the success of the hydrodynamic injection was variable and could only be assessed after visual inspection of hepatic sections. However, these results demonstrate that when injections are successful, the vast majority of co-injected constructs are co-localized within the same hepatocytes despite the disproportionate amounts of DNA that were injected. These results verified that multiple plasmids may be co-injected, efficiently transfected into hepatocytes, and co-expressed.

### Serum luciferase levels are an indicator of hepatocyte transfection efficiency and protein expression levels of co-injected constructs

**Figure 2:**
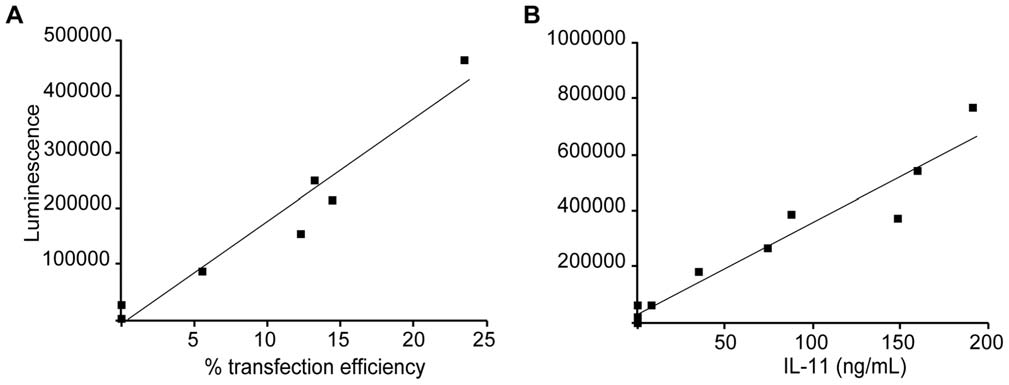
Serum luciferase activity is a surrogate marker for hepatocyte transfection efficiency and circulating serum levels of proteins encoded by co-transfected expression constructs. A. Serum luciferase levels and the percentage of transfected hepatocytes were determined in individual 6-week old ICR mice that had undergone hydrodynamic tail vein injection with GLuc (25 µg) and H2B-cherry (25 µg) expression plasmids 48 hours prior to analysis. **B.** Serum luciferase levels and IL-11 levels (r^2^ = 0.9293) were measured in 6-week old ICR mice that had undergone hydrodynamic tail vein injection with GLuc (25 µg) and IL-11 (25 µg) expression plasmids 48 hours prior to analysis.

The utility of hydrodynamic transfection is somewhat limited by the technique's variable transfection efficiency, which ranges from 0% to 25% (Fig. 2A). Currently, the transfection efficiency and hence the success of the injection cannot be easily determined. To overcome this limitation, we tested whether co-injection of an expression plasmid encoding secreted *Gaussia luciferase* (GLuc) would permit serum luciferase activity to serve as a surrogate marker for transfection efficiency and protein expression levels of co-injected genes. We elected to use GLuc because it has a short circulating half-life of ≈20 minutes and therefore will reflect short-term expression levels, is relatively non-toxic, and is robustly expressed and secreted by mammalian cells [24]. In addition, GLuc serum levels can be determined via a simple tail bleed without the need to sacrifice the animal. Thus, the use of GLuc would allow *in vivo* monitoring of gene expression over multiple time points. Unless otherwise indicated, we included an expression plasmid encoding SB100, a hyperactive transposase that leads to efficient integration of the *Sleeping Beauty* transposon [25], with all injections as it greatly increases the duration of GLuc expression from the transposable pT3 expression plasmid which was used for all experiments described herein (see Materials and Methods)(Fig. S1).

To test if serum GLuc activity is a reliable marker for hepatocyte transfection efficiency, we co-injected mice with 25 µg each of GLuc and H2B-cherry expression plasmids and subsequently measured the serum luciferase activity and determined hepatocyte transfection efficiency. The results of this experiment displayed a high degree of linear correlation (r^2^ = 0.93983) between the serum GLuc level and percent of H2B-cherry transfected hepatocytes (Fig. 2A). Next, we tested if serum GLuc levels might also serve as a reliable surrogate marker for circulating protein expression levels of co-injected constructs (Fig. 2B). Indeed, mice co-injected with GLuc and interleukin-11 (IL-11) plasmids displayed a high correlation (r^2^ = 0.9293) between serum GLuc and IL-11 levels (this correlation was also found in mice co-injected with GLuc and adiponectin, Fig. S2, r^2^ = 0.9849). These experiments also demonstrated that a threshold of serum luciferase activity (∼30,000 RLU) could be established to determine whether an animal had been successfully injected, as we saw very little hepatocyte transfection and protein expression when blood luciferase activity levels were below this level (Fig. 2A). Furthermore, we found that tail vein injection yielded supraphysiologic levels of protein expression. The endogenous circulating levels of IL-11 (undetectable; <5.56 ng/mL) and adiponectin (≈1–20 ng/mL) were markedly below those exhibited by successfully injected animals, generally greater than 100 ng/mL of either cytokine with successful injections (Fig. 2A, Fig. S2). Therefore, serum GLuc activity may be used to prospectively identify successful tail vein injections.

### Circulating levels of plasmid-encoded factors are influenced by the quantity and complexity of injected DNA

**Figure 3:**
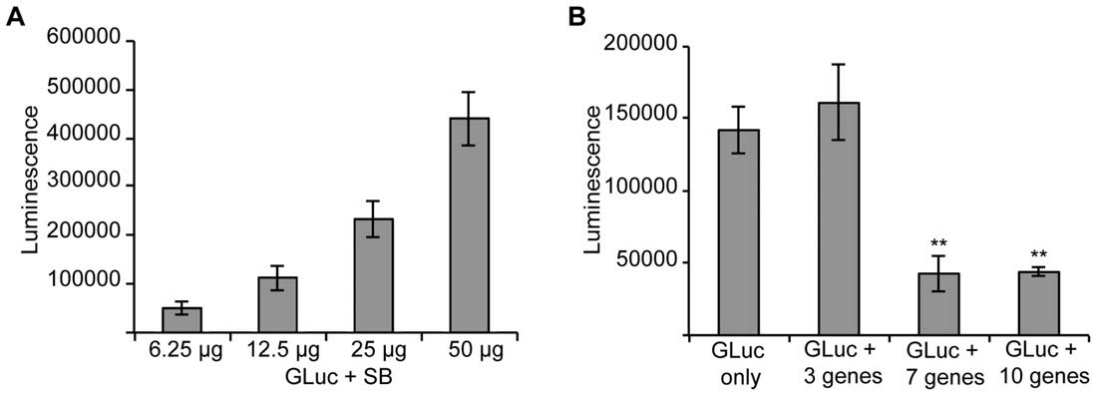
Serum luciferase activity is influenced by both the amount and the complexity of cDNA expression constructs that are transfected into liver cells via hydrostatic tail vein injection. A. Serum luciferase levels were measured 48 hours after 6-week old ICR mice (n = 5 animals per group) underwent hydrostatic tail vein injection with the indicated amount of the GLuc expression construct. **B.** Serum luiciferase levels were measured 48 hours after 6-week old ICR mice (n = 5 animals per group) underwent hydrostatic tail vein injection with GLuc (25 µg) and variable amounts (0, 75, 175 or 250 µg) of unrelated pT3 expression constructs. ** Indicates p<0.01 using a paired t-test.

Having established that serum GLuc activity is a useful surrogate marker for hepatocyte transfection efficiency and secreted protein expression levels, we sought to determine the minimum amount of DNA required for high levels of gene expression. To do this, mice were injected with increasing amounts of GLuc DNA and serum luciferase levels were measured 48 hours post-injection (Fig. 3A). At 25 µg and 50 µg of DNA per injection, luciferase levels were 6.5– and 16.5-fold higher than the cut-off of 30,000 RLU used to determine that an injection had failed (Fig. 2A and 2B). Since 25 µg of GLuc was the lowest amount of DNA we tested that reliably gave luciferase outputs several fold higher than the 30,000 RLU cut-off, was below saturating levels (further increase was still seen at 50 µg of DNA), and was a reasonable amount of DNA to prepare for experiments requiring multiple injections, we chose to utilize 25 µg of each ORF expression plasmid (pT3 ORF) for all injections.

Next, we sought to determine the number of individual genes that might be co-injected without significantly compromising the level of individual gene expression. To perform this experiment, mice were injected with GLuc alone or in combination with additional pT3 ORFs, and serum luciferase levels were measured 48 h post-injection (Fig. 3B). While luciferase levels were not significantly different between GLuc alone and GLuc + 3 genes (25 µg of each) (p = 0.59), they were significantly lower when 7 or 10 additional genes are added (p = 0.0017 and p = 0.0041, respectively). These results indicate that the maximum number of genes that may be co-injected with GLuc without compromising gene expression levels is between 3 and 6 genes. As described below, for purposes of screening we elected to maximize gene expression levels by co-injecting GLuc with only 3 additional ORFs selected from our expression library. Although our throughput would be increased by combining a higher number of ORFs, we felt that gene expression levels might be more variable once we initiated screening.

### Development of a moderate throughput in vivo screening protocol

**Figure 4:**
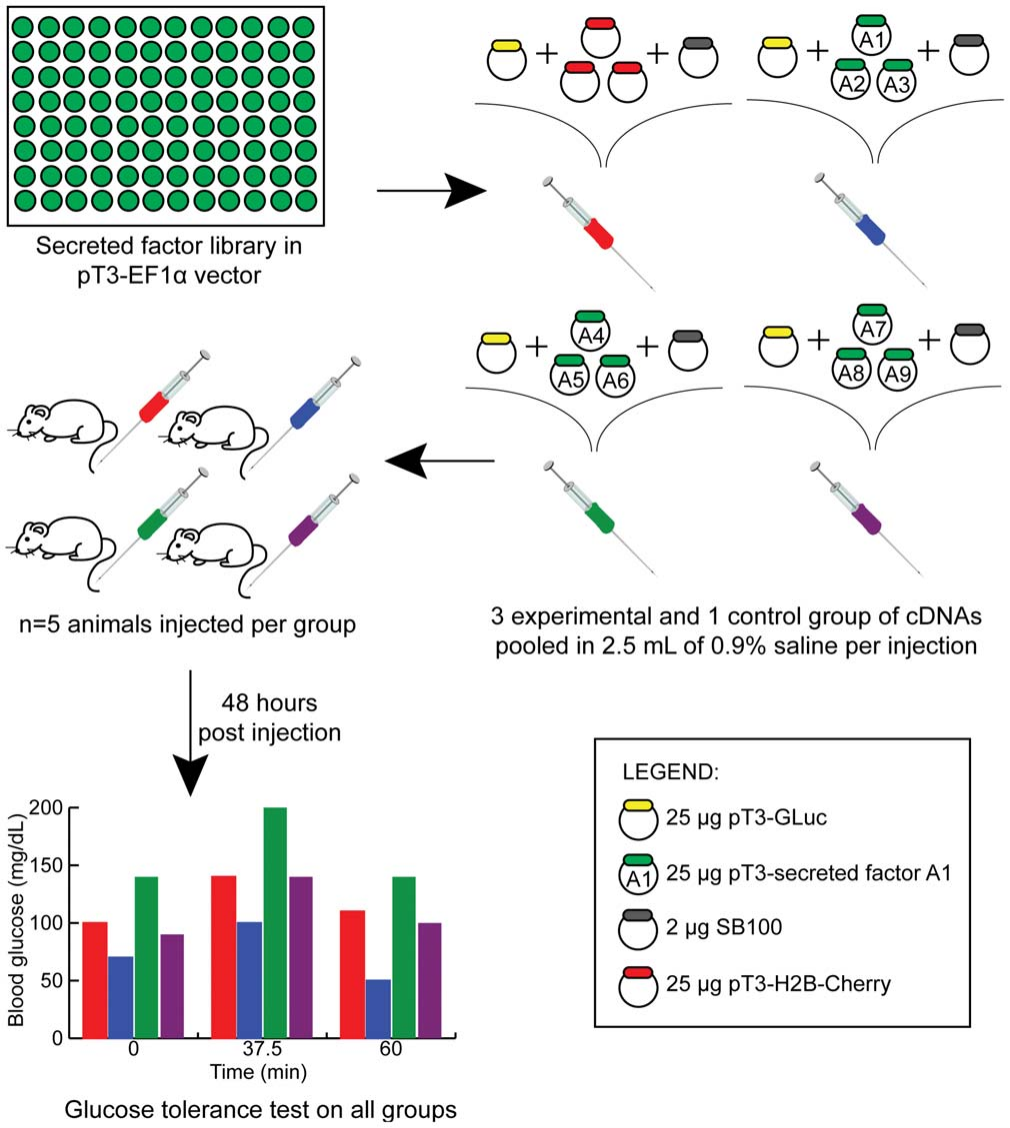
Development of an in vivo screening protocol. A Gateway ready arrayed library of 248 factors (left, top panel) were transferred into the transposable pT3 expression vector and isolated for injection. For each round of injection, a control DNA injection (GLuc (25 µg), SB100 (2 µg), and H2B-Cherry (75 µg)) and 3 experimental DNA injection formulations (GLuc (25 µg), SB100 (2 µg), and 3 secreted factors (25 µg each)) were prepared (right, top panel). Each cohort of 5 mice (aged 6–7 weeks) were injected with a single DNA formulation (left, middle panel). 43 h post-injection, mice were weighed and fasted. At 48 hours, animals underwent an i.p. glucose tolerance test with plasma glucose monitoring at times 0, 37.5 and 60 minutes. Subsequently, serum luciferase levels were determined to exclude mice with unsuccessful tail vein injections (luciferase values <30,000 relative light units). Sample results are illustrated in the left lower panel where the three potential outcomes are observed: improved glucose tolerance (blue formulation), worsened glucose tolerance (green formulation), and no effect (purple formulation).

To identify secreted factors that enhance glucose tolerance *in vivo*, we developed a moderate throughput screening protocol to screen our library containing a collection of randomly selected secreted factors (Fig. 4; Table S1, S2). In a typical experiment, 20 mice were distributed into four cohorts (1 control and 3 experimental groups). Since there was substantial variability in animal survival and injection success, any mouse exhibiting serum luciferase levels <30,000 RLU was excluded from analysis. If a cohort contained fewer than 3 viable mice, the experiment was repeated. All injections included the transposable GLuc and SB100 DNA vectors. Animals in the control group received 75 µg of pT3 H2B cherry DNA to match the total DNA delivered in the experimental cohorts, whereas animals in each experimental group received 3 secreted factors (25 µg each) selected from our pT3 ORF library of secreted factors. Thus, 9 secreted factors were screened in each round of injections. Two days post-injection, an i.p. glucose tolerance test (GTT) was performed. Gene combinations that were determined to be a primary hit, based upon a statistically significant change in glucose levels at any time point (0, 37.5 and 60 minutes) when compared with the control group, were re-tested individually i.e. each individual ORF was tested in a separate cohort of mice.

### IL-6 enhances glucose tolerance in euglycemic outbred mice

**Figure 5:**
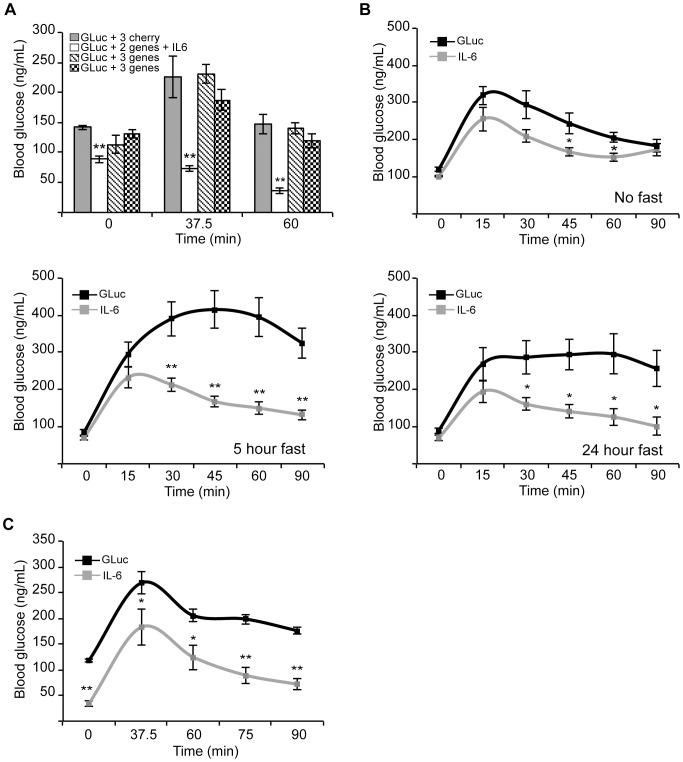
In vivo screening for secreted factors that enhance glucose tolerance identified interleukin-6. A. Glucose tolerance tests were performed on a cohort of mice (n = 4 animals per group) undergoing primary screening (1 control group and 3 experimental groups) for genes that modulate the glucose tolerance test. The second group contained the IL-6 ORF in addition to two other factors. ** Indicates p<0.01 using a paired t-test. **B.** Glucose tolerance tests were performed 48 hours post tail vein injection on 6 mice that received either GLuc (25 µg) and H2B-Cherry (25 µg), or GLuc and IL-6 (25 µg). Mice underwent no fast (right, upper panel, n = 5 animals per group), a 5 hour fast (left, lower panel, n = 5 animals per group) or an overnight fast (right, lower panel, n = 4 animals per group). *Indicates p<0.05 using a paired t-test. ** Indicates p<0.01 using a paired t-test. **C.** Glucose tolerance tests were performed 7 days post tail vein injection on mice that received either GLuc (25 µg) and H2B-cherry (25 µg), or GLuc and IL-6 (25 µg) (n = 5 animals per group). Animals were fasted for 5 hours prior to performing the GTT. *Indicates p<0.05 using a paired t-test. ** Indicates p<0.01 using a paired t-test.

Primary screening was performed on 248 genes. To our surprise, several factors previously implicated in glucose homeostasis such as adiponectin and resistin did not significantly alter the GTT (Fig. S3). By contrast, interleukin-6 (IL-6) was identified as a putative enhancer of glucose tolerance (Fig. 5A). Mice injected with IL-6 and two other secreted factors from the library exhibited significantly reduced blood glucose levels at 0, 37.5, and 60 minutes compared to the control (Fig. 5A).

Next, we confirmed the ability of IL-6 to improve glucose tolerance, in comparison to control injected animals, by performing GTTs under a variety of conditions (Fig. 5B). Compared to the control, IL-6 significantly improved glucose tolerance when the animals were not fasted, fasted for five hours, or fasted overnight. Furthermore, animals that expressed IL-6 continued to demonstrate improved glucose tolerance even one week after gene delivery (Fig. 5C). We note that animals that received IL-6 tended to have a lower fasting glucose.

### Several, but not all, IL-6 family members enhance glucose tolerance

**Figure 6:**
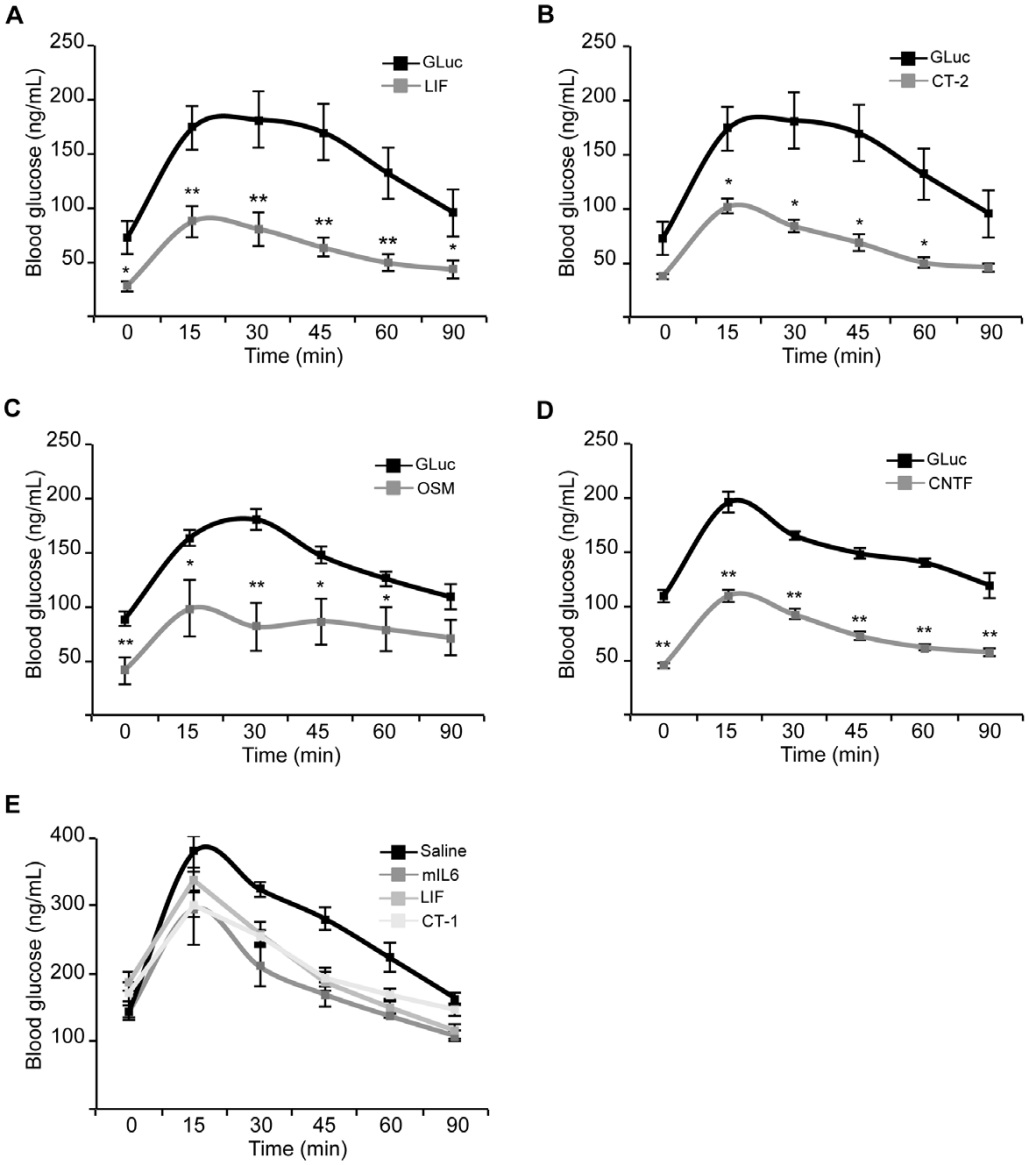
Several IL-6 family members enhance glucose tolerance. Glucose tolerance tests were performed 2 days post tail vein injection on mice that received either GLuc (25 µg) and H2B-cherry (25 µg), or: **A.** GLuc and LIF (25 µg) (n = 7 animals per group). **B.** GLuc and CT-2 (25 µg) (n = 7 animals per group). **C.** GLuc and OSM (25 µg) (n = 6 animals per group). **D.** GLuc and CNTF (25 µg) (n = 10 animals per group). All animals were fasted for 5 hours prior to the GTT. * Indicates p<0.05 using a paired t-test. ** Indicates p<0.01 using a paired t-test. **E.** Glucose tolerance testing was performed on 5-hour fasted animals that were injected i.p. with the indicated recombinant factor (n = 4) at time –30 minutes and then gavaged with a glucose solution (2g/kg) at time 0 minutes. Compared to saline treated animals, glucose values were significantly lower (p<0.04) for all experimentally treated animals at 15, 30, 45, 60 and 90 minutes (except CT-1 at 90 minutes).

IL-6 is a member of the IL-6 family of cytokines, which includes proteins such as leukemia inhibitory factor (LIF), cardiotrophin-2 (CT-2), oncostatin M (OSM), ciliary neurotrophic factor (CNTF), and interleukin-11 (IL-11). We tested six IL-6 family members for their ability to enhance glucose tolerance in our paradigm. Similar to IL-6, LIF, CT-2, OSM, and a secreted form of CNTF significantly enhanced glucose tolerance in wild-type mice (Fig. 6). In contrast, IL-11, CLC, and a non-secreted form of CNTF did not significantly improve the GTT (Fig. S4). Measurement of IL-11 levels in the blood of mice injected with an IL-11 encoding plasmid confirmed that elevated circulating levels of IL-11 were achieved but insufficient to improve glucose tolerance (Fig. 2). Therefore, several members of the IL-6 family are capable of improving glucose tolerance when expressed via hepatocyte transfection.

Prior work has demonstrated that a single dose of recombinant IL-6 or CT-1 improves glucose tolerance in mice [10], [26]. Our observation that hepatocyte expression of LIF improves glucose tolerance led us to test whether a single dose of recombinant LIF might have a similar effect (Figure 6E). Indeed, we found that treatment with recombinant LIF, similar to recombinant IL-6 and CT1, improves glucose tolerance. These findings validate the use of our *in vivo* screening paradigm as a platform to discover factors capable of modulating complex metabolic phenotypes such as glucose homeostasis.

## Discussion

Here we present the development and validation of an innovative *in vivo* screening platform to study the impact of systemic gene expression on complex metabolic parameters such as glucose homeostasis. This work makes several key advances on currently available techniques. First, we overcome the large expense and variable nature of available *in vivo* gene delivery techniques through the use of secreted *Gaussia luciferase* (GLuc). By measuring luciferase activity in the blood, successfully injected animals are identified prior to performing costly or time-consuming experiments and without the need to sacrifice the animals [20], [27]–[29]. Importantly, the use of an integrating transposable plasmid allows long-term expression studies to be performed [30]. Second, we have established that blood luciferase activity provides an indirect qualitative assessment of hepatocyte transfection efficiency and protein expression levels of co-transfected plasmids. Hence, dose-dependent effects are readily revealed. Third, we have established that multiple genes may be simultaneous screened *in vivo*. Fourth, we have used this screening platform to identify a previously unrecognized ability of several IL-6 family members (LIF, OSM and CT-2) to improve glucose tolerance. Finally, we confirmed the reliability of our experimental assay by demonstrating the ability of recombinant LIF to improve glucose tolerance. Thus, moderate throughput *in vivo* screening for inter-organ signaling factors that modulate metabolic parameters such as glucose homeostasis is made practical by the presented technique.

To validate the utility of our experimental method, we screened 248 secreted factors for the ability to improve glucose tolerance. We elected to study glucose tolerance because it exemplifies a disease-relevant biologically complex metabolic phenotype that integrates behavior and inter-organ homeostatic signaling that cannot be modeled *in vitro*. Interestingly, we found that IL-6 and several related family members not previously known to impact glucose homeostasis enhance the glucose tolerance of non-diabetic mice. In addition, we found that several factors previously shown to modulate glucose tolerance (FGF21, adiponectin, resistin, osteocalcin and IL1-RA) do not demonstrate activity in our assay despite achieving supraphysiologic levels in the blood [12], [15], [16], [31]–[34]. Although we suspect that this discrepancy reflects our use of non-diabetic animals, in contrast to the diabetic models used in prior studies, some of these factors have limited experimental evidence supporting their purported ability to directly influence glucose tolerance.

IL-6 is a pleitropic cytokine with a broad array of identified functions. These include promoting cellular proliferation, cellular differentiation, the inflammatory response, and the acute-phase response of multiple organ systems, including the skeletal, hematopoietic, and nervous systems [35]–[37]. Although the activity of IL-6 in glucose metabolism is controversial with results supporting both detrimental and beneficial impacts, evidence increasingly supports an advantageous role for IL-6. The hypothesized pathogenic role of IL-6 is largely based upon correlative data: T2DM is a chronic inflammatory state associated with both insulin resistance and elevated circulating levels of IL-6 [38]–[40]. Although some investigators have observed that IL-6 infusion causes hepatic insulin resistance [41], this view is challenged by the induction of IL-6 in response to insulin-sensitizing exercise [42] and the ability of IL-6 infusion to increase glucose uptake, glucose metabolism, and fat oxidation in isolated tissues *in vitro* as well as in diabetic and non-diabetic humans [43]–[45]. Additionally, mice that globally lack IL-6 or IL-6 receptor expression in the liver develop glucose intolerance [46], [47]. Indeed, recent work has demonstrated that exercise induced IL-6 expression enhances glucose tolerance by stimulating the expression of glucagon-like peptide-1 (GLP-1) from intestinal L cells and pancreatic α-cells [10]. Thus, our finding that hepatocyte IL-6 expression dramatically lowers circulating glucose levels at both time 0 and every time point up to 90 minutes in the GTT, up to one week post gene delivery is consistent with the potential beneficial metabolic activity of IL-6 (Fig. 5B, C, D).

IL-6 is a member of the IL-6 family of cytokines, which includes IL-11, LIF, OSM, CT-2, CLC, and CNTF. This cytokine family shares structural homology and signals through the gp130 receptor [48], [49] in conjunction with cytokine-selective co-receptors (IL-6R, IL-11R, LIFR, OSMR, CNTFR) [48]. Interestingly we found that the expression of several IL-6 family members (secreted CNTF, LIF, OSM, CT-2) but not others (IL-11, CLC, and a non-secreted version of CNTF) significantly improves glucose tolerance. These results suggest that the improved tolerance reflects activation of signaling through the common gp130, a close homolog of the leptin receptor. Although we are unsure what tissue or tissues are responsible for the enhanced tolerance we observe with IL-6 family members, the inactivity of IL-11 suggests that the target tissue does not express high levels of the IL-11 receptor or displays discrepant signaling in response to IL-11, IL-6 and other IL-6 family members that improve GTT. Given that loss of the hepatic IL-6 receptor expression is sufficient to cause glucose intolerance and that the IL-11 co-receptor is also expressed in this organ, we hypothesize that differential activation of gp130 signaling in the liver by IL-6 and IL-11 contributes to the enhanced glucose tolerance we observe in response to IL-6 but not IL-11 [47]. In addition, the ability of several IL-6 family members to improve glucose tolerance suggests that these factors could share the ability of IL-6 to stimulate L-cell and islet production of GLP-1.

The central contribution of this work is the development and demonstration of an *in vivo* method to rapidly screen for genetic factors that influence complex metabolic or behavioral phenotypes. Indeed, the recent identification of irisin highlights the potential value of an unbiased *in vivo* screening platform [50]. Irisin is an exercise-induced FNDC5 cleavage product that improves glucose tolerance by stimulating the metabolic activity of white fat. We suspect that other unexpected players in glucose homeostasis exist and remain to be discovered by the expanded use of our platform. While this report focused on glucose handling, our model is readily applicable towards screening for many different types of complex phenotypes such as hypercholesterolemia, weight maintenance, and tumor growth or metastasis that would be difficult to achieve otherwise.

## Materials and Methods

### Ethics Statement

Animals were housed and treated according to institutional guidelines. All animal protocols were approved by Stanford University's and Harvard University's Animal Care and Use Committees (Stanford Assurance #A3213–01; Harvard Assurance #A3593–01).

### Plasmids

A library of 182 mouse and 60 human fully sequenced open reading frames (ORFs), either known or predicted to be secreted, were obtained in the entry plasmid pDonor (CAT #12356–017) in an arrayed format (Invitrogen, Grand Island, NY). Individual ORFs were transferred into a Gateway ready transposable expression plasmid, T3-Ef1a vector (pT3), by LR recombination (Invitrogen, 1179–100). The pT3 vector and a high efficiency transposase expression plasmid were kindly provided by Aaron Tward and Zsuzsanna Izsvak. The secreted *Gaussia luciferase* gene (New England Biolabs, Ipswich, MA; CAT #N8082S) was subcloned into pEntr2B (Invitrogen, 11816–014) and then transferred into pT3 by LR recombination. IL-6 family members were synthesized in a Gateway ready format by GeneScript and transferred into the pT3 vector by LR recombination.

### Animal studies

6-week old female ICR mice weighing 20–25 g were obtained from Taconic (Germantown, NY) and Charles River (Wilmington, MA). Hydrodynamic tail injections were performed when animals reached 6–7 weeks of age. Mice were anesthetized via intraperitoneal (i.p.) injection with 0.3 mL of 1.2% Avertin solution. After adequate sedation was achieved, animals were placed under a 250 W heat lamp for 1 minute to promote vasodilatation and closely monitored for hyperthermia. Subsequently, the tail vein was cannulated with a 25G syringe and injected with 2.5 mL of normal saline (0.9%) solution containing 25 µg of each ORF being tested and 2 µg of the transposase over a total of 4–7 seconds. Typically, animals were injected in cohorts of 5 (total of 20 mice), and 3 independent ORFs were tested in each cohort. Conscious animals were returned to their cages without regard for the specific ORF they received to control for cage-specific effects.

### Glucose tolerance test

For experiments using the hydrodynamic tail vein injection: 43 hours post-injection, mice were weighed and food was withdrawn. Water was available at all times. After five hours, a time 0 blood glucose measurement was made and mice were injected i.p. with 2g/kg of D-glucose solution in distilled water (time 0). Blood glucose levels were subsequently taken at times 37.5, 60, and 90 minutes (unless otherwise indicated in text) by nicking the tail and collecting a droplet of blood on a OneTouch Ultra 2 glucometer (LifeScan, Milpitas, CA).

For experiments using recombinant protein: 6-week old ICR mice were fasted for 5 hours and then i.p. injected with vehicle, LIF (2.5 µg/mouse; Fisher Scientific #5057119,), IL-6 (1.25 µg/mouse; R&D Systems #406-ML-005) or CT-1 (2.5 µg/mouse; Fisher Scientific #50934512) in groups of four (16 animals total). After 30 minutes, blood glucose levels were measured (time 0) and animals were gavaged with 2g/kg of D-glucose solution in distilled water. Subsequent glucose measurements were preformed as described above.

### Analysis of serum protein expression levels

0.5 mL of blood was collected from anesthetized mice (1 mL of 1.2% Avertin) by retro-orbital bleeding (VWR, 14705–003). Blood samples were centrifuged at 2500 rpm for 5 minutes at 4°C and the serum was collected for protein expression analysis. IL-6 (Invitrogen, KHC0061), IL-11 (Abnova, KA1792), and adiponectin (Invitrogen, KRP0041) ELISA kits were used in accordance with the manufacturer's instructions. Sample dilutions were performed until all values were within the linear range of the assay.

Serum luciferase activity was performed using the GLuc assay kit (New England Biolabs, CAT #E3300L). Briefly, 10 µL of serum was obtained by either tail or retro-orbital bleed, mixed with 50 µL of substrate by automated injection, and measured using a LUMIstar Optima luminometer (BMG Labtech, Cary, NC) using a delay of 0.2 s, an integration time of 1.5 s, and a gain of 3500. To standardize luciferase measurements taken across time, a 10 µL aliquot of conditioned media collected from GLuc transfected HEK 293T cells was performed with each assay.

### Histological procedures and fluorescence visualization

To determine hepatic transfection efficiency, livers were harvested from mice 48 hours after mice had received pT3-H2B-cherry and/or pT3-H2B-GFP expression vectors. The livers were fixed in 4% paraformaldehyde for 1.5 hours and sunk overnight in 30% sucrose solution. 12 micron sections were cut and subjected to Hoechst staining to identify cell nuclei. Cell counting was performed using the cell detection algorithm in Bitplane Imaris, and transfection efficiency was determined by dividing the number of red, green or double positive cells by the total cell nuclei number.

### Statistics

Significance of results was determined using a standard 2-tailed Student's *t* test. Error bars show standard error of the mean unless otherwise noted. All presented experiments were repeated at least twice.

## Supporting Information

Figure S1 **Serum luciferase activity correlates with circulating serum levels of proteins encoded by co-transfected expression constructs.** Serum luciferase levels and adiponectin levels (r^2^ = 0.9849) were measured in 6-week old ICR mice that had undergone hydrodynamic tail vein injection with GLuc (25 µg) and adiponectin (25 µg) expression plasmids 48 hours prior to analysis.
(TIF)

Figure S2 **Addition of SB100 extends luciferase duration. A.** Serum luciferase measurements were made at various time points after tail vein injection of 6-week old ICR mice with 25 µg of a GLuc transposable expression plasmid (n = 8 animals) or no GLuc expression plasmid as a negative control (n = 1). **B.** Serum luciferase measurements were made at various time points after tail vein injection as in A, with the addition of 2 µg of SB100.
(TIF)

Figure S3 **Previously identified adipocyte-secreted hormones adiponectin and resistin do not impact glucose tolerance. A.** Glucose tolerance tests were performed 48 hours post tail vein injection on mice that received either GLuc (25 µg) and H2B-cherry (25 µg), or GLuc and adiponectin (25 µg) (n = 5 animals per group). Animals were fasted for 5 hours prior to performing the GTT. **B.** Glucose tolerance tests were performed 48 hours post tail vein injection on mice that received either GLuc (25 µg) and H2B-cherry (25 µg), or GLuc and resistin (25 µg) (n = 5 animals per group). Animals were fasted for 5 hours prior to performing the GTT.
(TIF)

Figure S4 **Several IL-6 family members do not enhance glucose tolerance.** Glucose tolerance tests were performed 2 days post tail vein injection on mice that received either GLuc (25 µg) and H2B-cherry (25 µg), or: **A.** GLuc and IL-11 (25 µg) (n = 9 animals per group). **B.** GLuc and CLC (25 µg) (n = 9 animals per group). **C.** GLuc and a non-secreted version of CNTF (25 µg) (n = 7 animals per group).
(TIF)

Table S1 **Mouse secreted factors tested for the ability to enhance glucose tolerance.**
(XLSX)

Table S2 **Human secreted factors tested for the ability to enhance glucose tolerance.**
(XLSX)
