# Correction: *In Vivo* Screening for Secreted Proteins That Modulate Glucose Handling Identifies Interleukin-6 Family Members as Potent Hypoglycemic Agents

**DOI:** 10.1371/annotation/6e392eb7-55cb-4bc6-ab8e-bd8130ad6582

**Published:** 2012-11-20

**Authors:** Chen Amy Chen, Peter C. Carolan, Justin P. Annes

There was an error in the second author's name. Peter J. Carolan is correct. The correct citation is: Chen CA, Carolan PJ, Annes JP (2012) In Vivo Screening for Secreted Proteins That Modulate Glucose Handling Identifies Interleukin-6 Family Members as Potent Hypoglycemic Agents. PLoS ONE 7(9): e44600. doi:10.1371/journal.pone.0044600 

