# Supplementary figures and images for: In Vivo Screening for Secreted Proteins That Modulate Glucose Handling Identifies Interleukin-6 Family Members as Potent Hypoglycemic Agents
Source: PLoS One. 2012 Sep 4;7(9):e44600. doi: 10.1371/journal.pone.0044600 (PMC3433445; doi:10.1371/journal.pone.0044600)

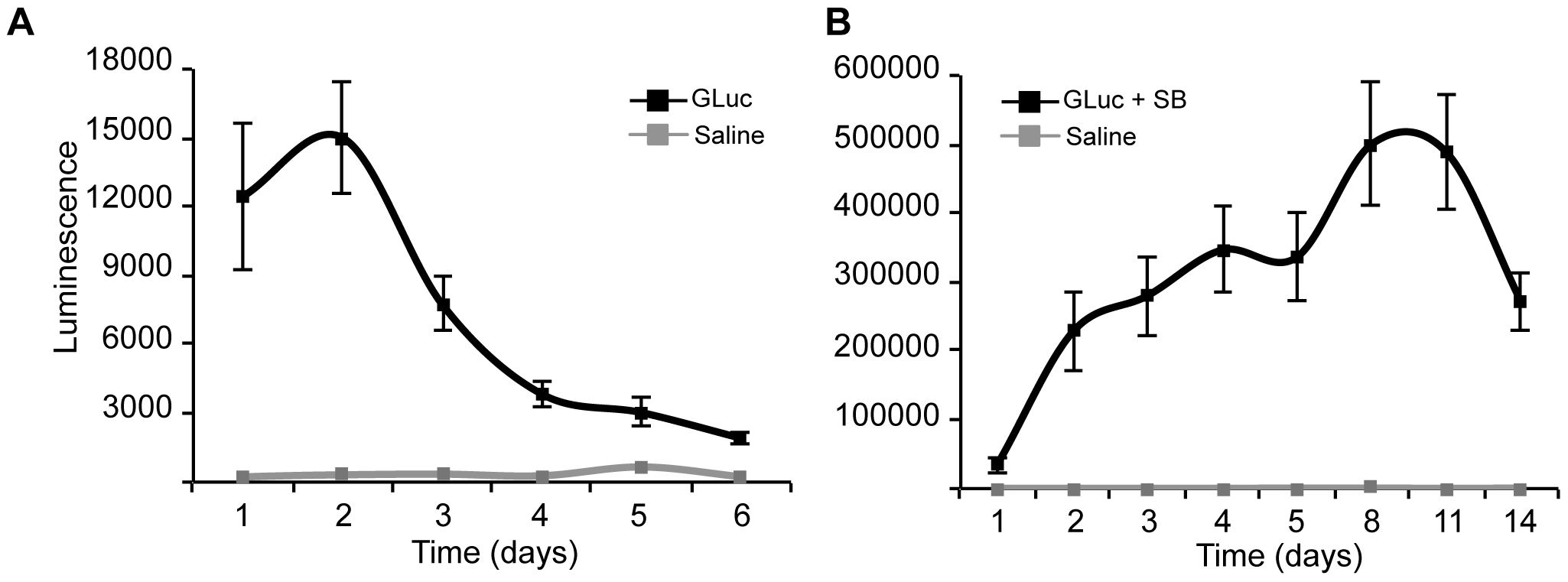

Supplement: Figure S1 — Serum luciferase activity correlates with circulating serum levels of proteins encoded by co-transfected expression constructs. Serum luciferase levels and adiponectin levels (r2 = 0.9849) were measured in 6-week old ICR mice that had undergone hydrodynamic tail vein injection with GLuc (25 µg) and adiponectin (25 µg) expression plasmids 48 hours prior to analysis. (TIF) [file pone.0044600.s001.tif]

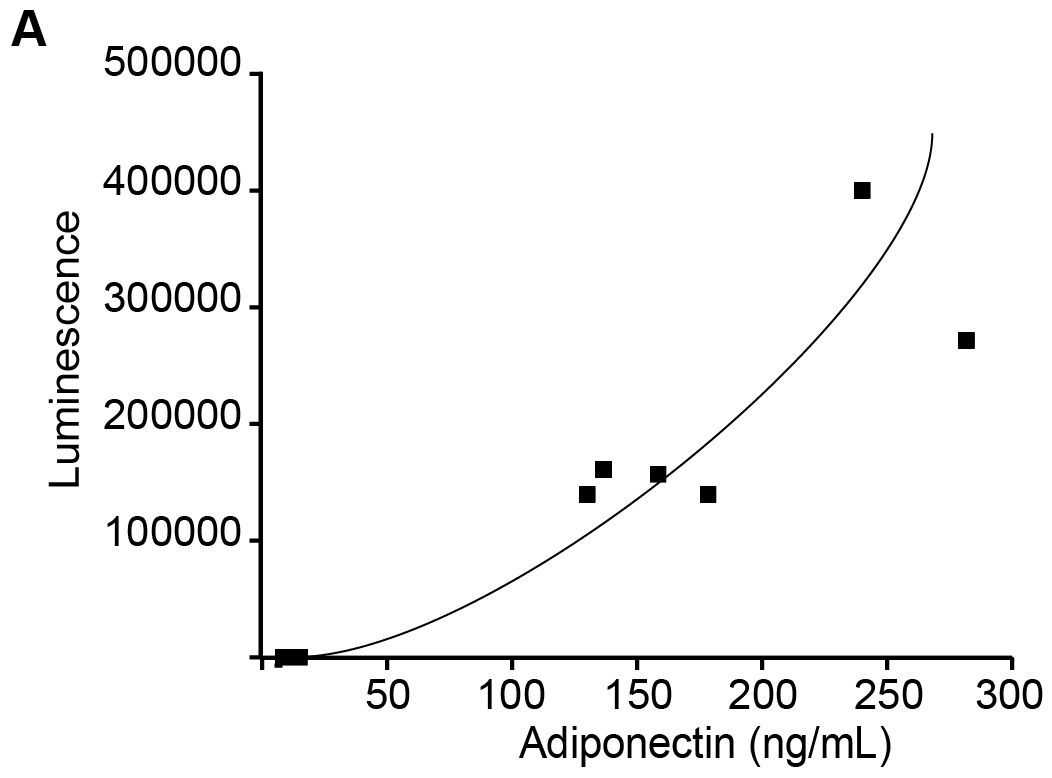

Supplement: Figure S2 — Addition of SB100 extends luciferase duration. A. Serum luciferase measurements were made at various time points after tail vein injection of 6-week old ICR mice with 25 µg of a GLuc transposable expression plasmid (n = 8 animals) or no GLuc expression plasmid as a negative control (n = 1). B. Serum luciferase measurements were made at various time points after tail vein injection as in A, with the addition of 2 µg of SB100. (TIF) [file pone.0044600.s002.tif]

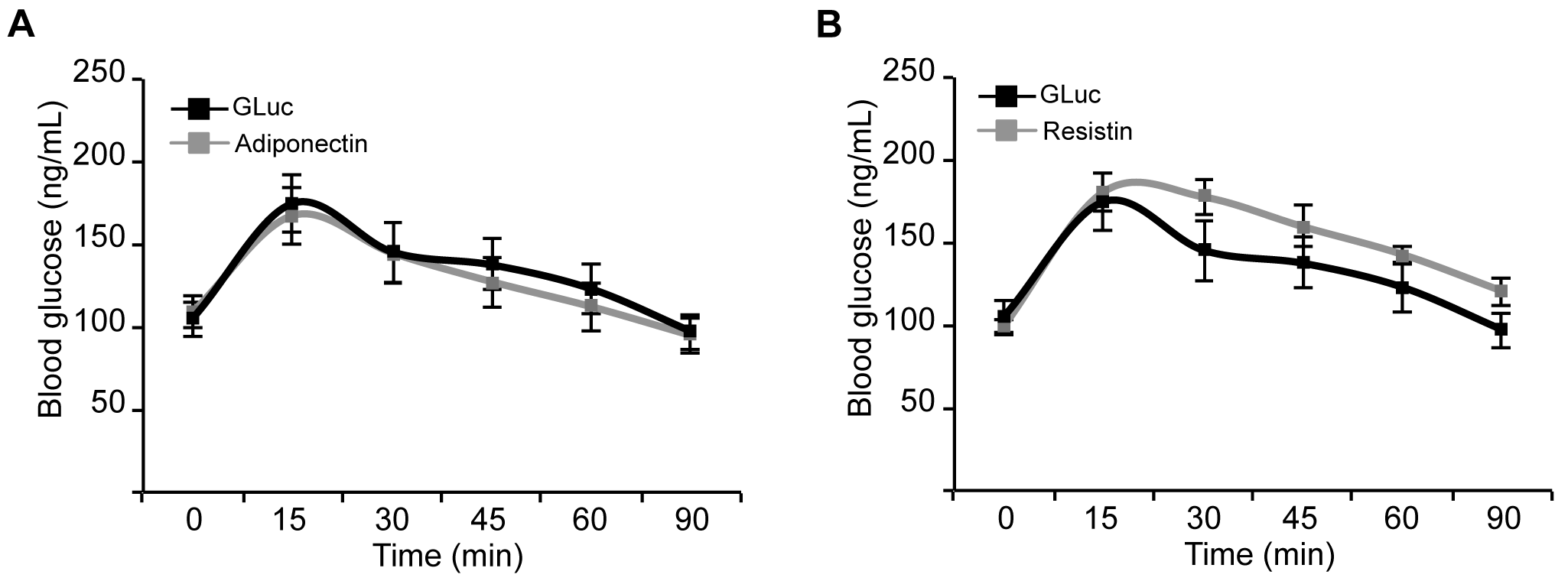

Supplement: Figure S3 — Previously identified adipocyte-secreted hormones adiponectin and resistin do not impact glucose tolerance. A. Glucose tolerance tests were performed 48 hours post tail vein injection on mice that received either GLuc (25 µg) and H2B-cherry (25 µg), or GLuc and adiponectin (25 µg) (n = 5 animals per group). Animals were fasted for 5 hours prior to performing the GTT. B. Glucose tolerance tests were performed 48 hours post tail vein injection on mice that received either GLuc (25 µg) and H2B-cherry (25 µg), or GLuc and resistin (25 µg) (n = 5 animals per group). Animals were fasted for 5 hours prior to performing the GTT. (TIF) [file pone.0044600.s003.tif]

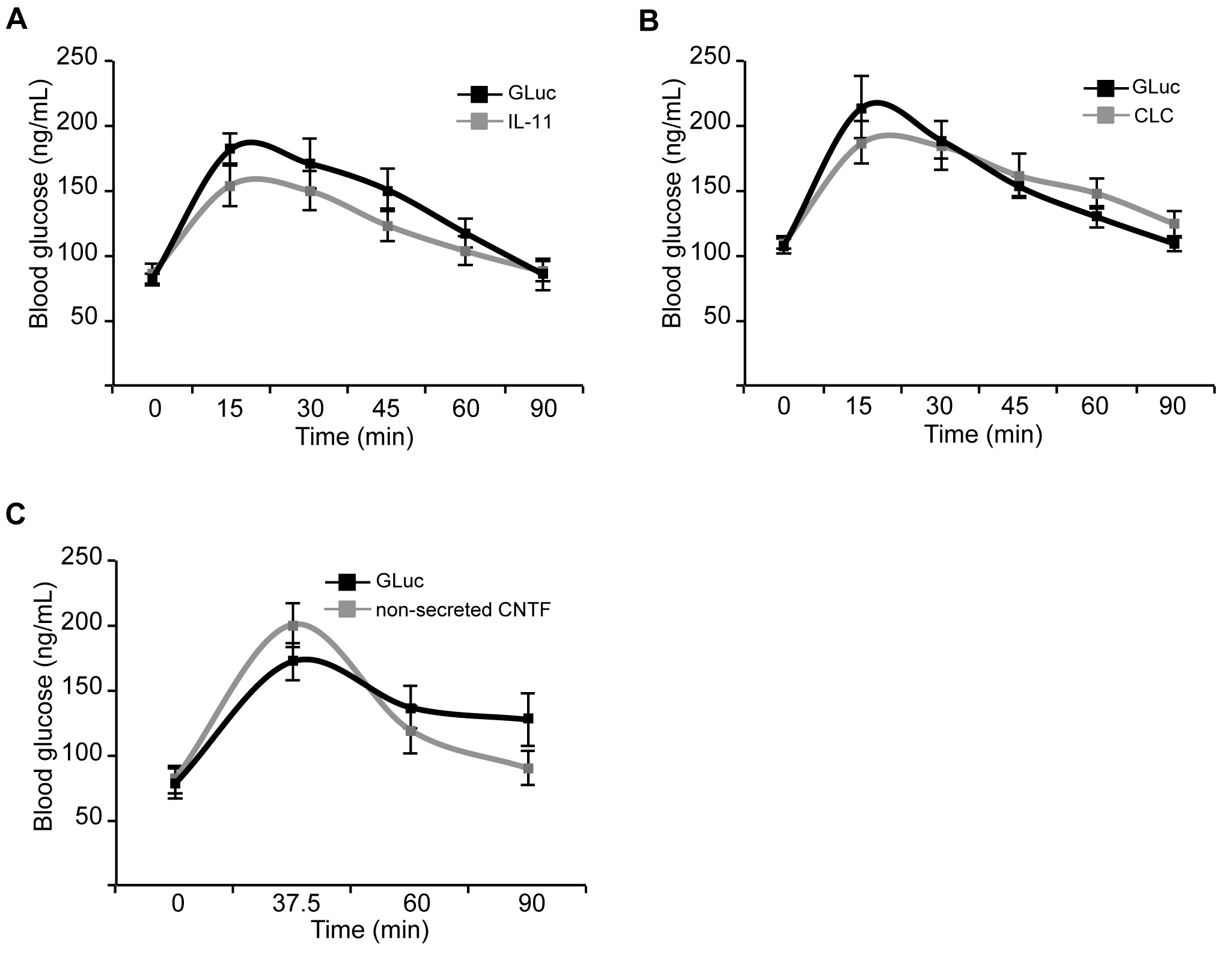

Supplement: Figure S4 — Several IL-6 family members do not enhance glucose tolerance. Glucose tolerance tests were performed 2 days post tail vein injection on mice that received either GLuc (25 µg) and H2B-cherry (25 µg), or: A. GLuc and IL-11 (25 µg) (n = 9 animals per group). B. GLuc and CLC (25 µg) (n = 9 animals per group). C. GLuc and a non-secreted version of CNTF (25 µg) (n = 7 animals per group). (TIF) [file pone.0044600.s004.tif]
